# Supplementary material for: Maternal Fever and Reduced Fetal Movement as Predictive Risk Factors for Adverse Neonatal Outcome in Cases of Congenital SARS-CoV-2 Infection: A Meta-Analysis of Individual Participant Data from Case Reports and Case Series
Source: Viruses. 2023 Jul 24;15(7):1615. doi: 10.3390/v15071615 (PMC10384546; doi:10.3390/v15071615)
Supplement: Supplementary file 1 [file viruses-15-01615-s001.zip › viruses-2492773-supplementary.pdf]

Quality Assessment and Summary of Key Characteristics of Included Studies

Table S1. Case Reports/Case Series Studies assessed using the Murad Tool Scoring Criteria [1]. The study is classified as "good quality" – score 4, "fair quality" – score 3 and "poor quality" score between 1-2

**Author**	**Country**	**Number of eligible newborns**	**Quality Assessment Score**
**Abadía-Cuchí *et al* [2]**	Spain	1	2
**Alzamora MC *et al* [3]**	Peru	1	4
**Babal *et al* [4]**	Slovakia	1	2
**Boncompagni *et al*[5]**	Italy	1	3
**Bouachba[6]**	France	2	4
**Buonsenso D *et al*[7]**	Italy	1	3
**Bullok et al [8]**	USA	1	4
**Carvalho *et al*[9]**	Brazil	2	4
**Choobdar *et al*[10]**	Iran	1	3
**Correia *et al*[11]**	Portugal	1	2
**Daza[12]**	Columbia	1	4
**Disse *et al*[13]**	Germany	2	3
**Dong *et al*[14]**	China	1	4
**Enache *et al*[15]**	Romania	1	3
**Eich[16]**	Germany	1	4
**Ergon *et al*[17]**	Turkey	1	3
**Facchetti *et al*[18]**	Italy	1	2
**Farhadi *et al*[19]**	Iran	1	4
**Favre *et al*[20]**	Switzerland	1	3
**Fitzgerald *et al*[21]**	Ireland	5	3
**Fusco *et al*[22]**	Brazil	3	4
**Gant *et al*[23]**	USA	1	4
**Garcia-Ruiz *et al*[24]**	Spain	1	2
**Guan[25]**	USA	1	4
**Karade *et al*[26]**	India	1	3
**Kirtsman *et al*[27]**	Canada	1	3
**Kohorn *et al*[28]**	USA	1	3
**Lesieur *et al*[29]**	France	1	3

**Lorenz** ***N et al*****[30]**	Germany	1	3
**Marinho** ***et al*****[31]**	Brazil	1	4
**Marton** ***et al*****[32]**	UK	1	2
**Marzollo** ***et al*****[33]**	Italy	1	3
**Morales et al[34]**	Mexico	1	3
**Min Yi et al[35]**	Singapore	1	2
**NG David** ***et al*** **[36]**	Malaysia	1	3
**Parsa** ***et al*****[37]**	Iran	1	4
**Popescu** ***et al*****[38]**	Romania	1	2
**Pulinx** ***et al*****[39]**	Belgium	2	2
**Reagan-Steiner** ***et al*****[40]**	USA	1	3
**Rebello** ***et al*****[41]**	Brazil	1	3
**Richtmann** ***et al*****[42]**	Brazil	1	4
**Rodrigues** ***et al*****[43]**	Portugal	1	2
**Sagara et al[44]**	Japan	1	4
**Schwartz[45]**	Georgia	2	2
**Sessa** ***et al*** **[46]**	Italy	2	3
**Shaiba** ***et al*****[47]**	Saudi-Arabia	1	2
**Shen** ***et al*****[48]**	USA	1	2
**Shook** et al**[49]**	USA	2	4
**Sukhikh** ***et al*****[50]**	Russia	1	3
**Vivanti** ***et al*****[51]**	France	5	2
**Vivanti** ***et al*****[52]**	France	1	4
**Zaigham** ***et al*****[53]**	Sweden	9	3
**Zamaniyan** ***et al*****[54]**	Iran	1	4
**Zhang** ***et*** **[55]**	USA	1	2
**Zinserling** ***et al*****[56]**	Russia	1	4

Table S2. Scoring Criteria for the Murad Tool

**Selection**	• Were the cases presented from a particular study period?
**Ascertainment**	• Were the cases positive for COVID-19 according to the accepted criteria?<br>• Was the neonates' clinical status reported?
**Causality**	• Have alternative causes for the presence of symptoms besides COVID-19 infection been ruled out?<br>• Was the duration of the follow-up sufficient for outcomes to occur, such as neonatal discharge or death?

**Reporting**	• Was the case described sufficiently for other researchers to replicate the study or for practitioners to draw inferences about their own practice?

Table S3. Studies were assessed based on Newcastle-Ottawa Scale. A study with a score of 6 or more is classified as high quality, 4-5 as medium quality, and less than 4 as low quality, using the Newcastle-Ottawa Scale[57].

Study	**Country**	**Cohort size**	**Number of cases with confirmed vertical transmission**	**Quality Assessment Score**
Argueta *et al*[58]	USA	54	1	5
Fenizia *et al* [59]	Italy	31	1	5
Garrido-Pontnou *et al* [60]	Spain	198	4	2

Table S4. Scoring Criteria for the Newcastle-Ottawa Scale

**Selection**	• Were the cases presented representative of the average in the target population?<br>• Were there cases with negative COVID-19 test?<br>• Were the cases taken from medical records?<br>• Was the subject of vertical transmissions followed in the study from de beginning?
**Comparability**	• Were the cases of interest compared to other groups based on the study design or analysis?
**Outcome**	• Was there sufficient clinical data on the neonates?<br>• Were the confirmed cases followed up long enough for outcome to occur?<br>• Was there a complete follow-up of the confirmed cases?

Reference

1. M. H. Murad, S. Sultan, S. Haffar, and F. Bazerbachi, "Methodological quality and synthesis of case series and case reports," *BMJ Evid Based Med*, vol. 23, no. 2, pp. 60–63, Apr. **2018**, doi: 10.1136/bmjebm-2017-110853.
2. N. Abadía-Cuchí, N.; Ruiz-Martínez, S.; Fabre, M.; Mateo, P.; Remacha Sienes, M.; Ventura Faci, P.; Bueno Sancho, J.; Paules, C, "SARS-CoV-2 congenital infection and pre-eclampsia-like syndrome in dichorionic twins: A case report and review of the literature," *Int J Gynaecol Obstet*, vol. 154, no. 2, pp. 370–372, Aug. **2021**, doi: 10.1002/ijgo.13749.
3. M. C. Alzamora, T. Paredes, D. Caceres, C. M. Webb, L. M. Valdez, and M. La Rosa, "Severe COVID-19 during Pregnancy and Possible Vertical Transmission," *Am J Perinatol*, vol. 37, no. 8, pp. 861–865, Jun. **2020**, doi: 10.1055/s-0040-1710050.
4. Babal, P.; Krivosikova, L.; Sarvaicova, L.; Deckov, I.; Szemes, T.; Sedlackova, T.; Palkovic, M.; Kalinakova, A.; Janega, P, "Intrauterine Fetal Demise After Uncomplicated COVID-19: What Can We Learn from the Case?," *Viruses*, vol. 13, no. 12, p. 2545, Dec. **2021,** doi: 10.3390/v13122545.

5. Boncompagni, A.; De Agostini, M.; Lugli, L.; Ternelli, G.; Colonna, V.; Biagioni, E.; Bonasoni, M.P.; Salviato, T.; Gabrielli, L.;Falconi, M.; et al, "Unexpected Vertical Transmission of SARS-CoV-2: Discordant Clinical Course and Transmission from Mother to Newborn," *Microorganisms*, vol. 10, no. 9, p. 1718, Aug. **2022**, doi: 10.3390/microorganisms10091718.
6. A. Bouachba *et al.*, "Placental lesions and SARS-Cov-2 infection: Diffuse placenta damage associated to poor fetal outcome," *Placenta*, vol. 112, pp. 97–104, Sep. **2021**, doi: 10.1016/j.placenta.2021.07.288.
7. Buonsenso, D.; Costa, S.; Sanguinetti, M.; Cattani, P.; Posteraro, B.; Marchetti, S.; Carducci, B.; Lanzone, A.; Tamburrini, E.; Vento, G.; Valentini, P, "Neonatal Late Onset Infection with Severe Acute Respiratory Syndrome Coronavirus 2," *Am J Perinatol*, vol. 37, no. 8, pp. 869–872, Jun. **2020,** doi: 10.1055/s-0040-1710541.
8. Bullock, H. A.; Fuchs, E.; Martines, R. B.; Lush, M.; Bollweg, B.; Rutan, A.; Nelson, A.; Brisso, M.; Owusu-Ansah, A.; Sitzman, C,"Probable vertical transmission of Alpha variant of concern (B.1.1.7) with evidence of SARS-CoV-2 infection in the syncytiotrophoblast, a case report," *Front Med (Lausanne)*, vol. 9, p. 1099408, Jan. **2023**, doi: 10.3389/fmed.2022.1099408.
9. Ferreira, M.D.F.C.; Pavon, J.A.R.; Napoleão, A.C.B.; Figueiredo, G.M.D.P.; Florêncio, P.C.B.; Arantes, R.B.d.S.; Rizzo, P.S.; Carmo, M.A.M.V.; Nakazato, L.; Dutra, V.; et al., "Clinical and genomic data of sars-cov-2 detected in maternal-fetal interface during the first wave of infection in Brazil," *Microbes Infect*, vol. 24, no. 4, p. 104949, Jun. **2022**, doi: 10.1016/j.micinf.2022.104949.
10. Choobdar, F.A.; Ghassemzadeh, M.; Attarian, M.; Abbariki, E.; Nateghian, A.; Ghanbari, B.; Hamzehi, S.S.; Hashemi, M.R.; Azarbin, Z., "Transplacental Transmission of SARS-CoV-2 Infection: A Case Report from Iran," *Arch Pediatr Infect Dis*, vol. 9, no. 2, Art. no. 2, **2021**, doi: 10.5812/pedinfect.108582.
11. Correia, C.R.; Marçal, M.; Vieira, F.; Santos, E.; Novais, C.; Maria, A.T.; Malveiro, D.; Prior, A.R.; Aguiar, M.; Salazar, A.; et al., "Congenital SARS-CoV-2 Infection in a Neonate With Severe Acute Respiratory Syndrome," *Pediatr Infect Dis J*, vol. 39, no. 12, pp. e439–e443, Dec. **2020**, doi: 10.1097/INF.0000000000002941.
12. Daza, M.; Corchuelo, S.; Osorio, J.; Alberto Gómez, L.; Parra, E.; Alarcón, Á.; Mercado, M., "Fetal demise and SARS-CoV-2 infection during pregnancy: Histopathological and immunohistochemical findings of three cases referred to the Colombian National Institute of Health," *Clinical Infection in Practice*, vol. 17, p. 100219, Jan. **2023**, doi: 10.1016/j.clinpr.2023.100219.
13. Disse, S.C.; Manuylova, T.; Adam, K.; Lechler, A.; Zant, R.; Klingel, K.; Aepinus, C.; Finkenzeller, T.;Wellmann, S.; Schneble, F., "COVID-19 in 28-Week Triplets Caused by Intrauterine Transmission of SARS-CoV-2-Case Report," *Front Pediatr*, vol. 9, p. 812057, **2021**, doi: 10.3389/fped.2021.812057.
14. Dong, L.; Tian, J.; He, S.; Zhu, C.; Wang, J.; Liu, C.; Yang, J., "Possible Vertical Transmission of SARS-CoV-2 From an Infected Mother to Her Newborn," *JAMA*, vol. 323, no. 18, pp. 1846–1848, May **2020**, doi: 10.1001/jama.2020.4621.
15. Enache, A.; Ciocan, V.; Muresan, C.O.; Cut, T.G.; Novacescu, D.; Paul, C.; Andreescu, N.; Mihailescu, A.; Raica, M.; Dumache, R., "Postmortem Documentation of SARS-CoV-2 in Utero and Postpartum Transmission, through Amniotic Fluid, Placental, and Pulmonary Tissue RT-PCR," *Applied Sciences*, vol. 11, no. 20, Art. no. 20, Jan. **2021**, doi: 10.3390/app11209505.
16. Kienast, P.; Prayer, D.; Binder, J.; Prayer, F.; Dekan, S.; Langthaler, E.; Sigl, B.; Eichinger, S.; Perkmann-Nagele, N.; Stuempflen, I. et al., "SARS-CoV-2 variant-related abnormalities detected by prenatal MRI: a prospective case–control study," *The Lancet Regional Health – Europe*, vol. 0, no. 0, Jan. 2023, doi: 10.1016/j.lanepe.2023.100587.
17. Ergon, E.Y.; Akbay, S.; Aytemiz, G.; Çelik, E.C.A.; Çalıskan Polat, A.; Umit, Z.; Paytoncu, S., "A novel case of neonatal acute respiratory distress syndrome with SARS-CoV-2 infection: potential perinatal transmission," *Arch Argent Pediatr*, vol. 119, no. 5, pp. e531–e535, Oct. 2021, doi: 10.5546/aap.2021.eng.e531.
18. Facchetti, F.; Bugatti, M.; Drera, E.; Tripodo, C.; Sartori, E.; Cancila, V.; Papaccio, M.; Castellani, R.; Casola, S.; Boniotti, M.B.; et al., "SARS-CoV2 vertical transmission with adverse effects on the newborn revealed through integrated immunohistochemical, electron microscopy and molecular analyses of Placenta," *EBioMedicine*, vol. 59, p. 102951, Sep. 2020, doi: 10.1016/j.ebiom.2020.102951.
19. Farhadi, R.; Mehrpisheh, S.; Ghaffari, V.; Haghshenas, M.; Ebadi, A., "Clinical course, radiological findings and late outcome in preterm infant with suspected vertical transmission born to a mother with severe COVID-19 pneumonia: a case report," *J Med Case Rep*, vol. 15, no. 1, p. 213, Apr. 2021, doi: 10.1186/s13256-021-02835-0.

20. Favre, G.; Mazzetti, S.; Gengler, C.; Bertelli, C.; Schneider, J.; Laubscher, B.; Capoccia, R.; Pakniyat, F.; Ben Jazia, I.; Eggel-Hort, B.; et al., "Decreased Fetal Movements: A Sign of Placental SARS-CoV-2 Infection with Perinatal Brain Injury," *Viruses*, vol. 13, no. 12, p. 2517, Dec. 2021, doi: 10.3390/v13122517.
21. Fitzgerald, B.; O'Donoghue, K.; McEntagart, N.; Gillan, J.E.; Kelehan, P.; O'Leary, J.; Downey, P.; Dean, J.; De Gascun, C.F, B., "Fetal Deaths in Ireland Due to SARS-CoV-2 Placentitis Caused by SARS-CoV-2 Alpha," *Arch Pathol Lab Med*, vol. 146, no. 5, pp. 529–537, May 2022, doi: 10.5858/arpa.2021-0586-SA.
22. Fusco, M.A.; Mantini, V.; de Souza Salmont Júnior, J.; de Carvalho Gomes, R.G.; de Oliveira Lima, A.R.; de Oliveira Clarim, H.L.; Ferreira, E.C.; de Abreu Almeida, S.S.; Salmont, C.G.; de Sousa Rizzo-Valente, V.; et al. "Assessment of SARS-CoV-2 Vertical Transmission through Nested RT-PCR Testing of Neonatal Samples: Three Case Reports," *Journal of Pediatrics, Perinatology and Child Health*, vol. 6, no. 3, pp. 370–376, Sep. 2022.
23. T. F. Gant, T. P. Villegas, J. Summerall-Smith, and B. Watkins, "Intrauterine fetal demise as a result of maternal COVID-19 infection in the third trimester of pregnancy: A case report," *Int J Surg Case Rep*, vol. 98, p. 107492, Sep. 2022, doi: 10.1016/j.ijscr.2022.107492.
24. Garcia-Ruiz, I.; Sulleiro, E.; Serrano, B.; Fernandez-Buhigas, I.; Rodriguez-Gomez, L.; Sanchez-Nieves Fernandez, D.; Anton-Pagarolas, A.; Esperalba-Esquerra, J.; Frick, M. A.; Camba, F.; et al. "Congenital infection of SARS-CoV-2 in live-born neonates: a population-based descriptive study," *Clin Microbiol Infect*, vol. 27, no. 10, p. 1521.e1-1521.e5, Oct. **2021**, doi: 10.1016/j.cmi.2021.06.016
25. Guan, M.; Johannesen, E.; Tang, C. Y.; Hsu, A. L.; Barnes, C. L.; Burnam, M.; McElroy, J. A.; Wan, X.-F., "Intrauterine Fetal Demise in the Third Trimester of Pregnancy Associated With Mild Infection With the SARS-CoV-2 Delta Variant Without Protection From Vaccination," *J Infect Dis*, vol. 225, no. 5, pp. 748–753, Mar. **2022**, doi: 10.1093/infdis/jiac007.
26. S. Karade, A. K. Vishal, S. Sen, N. Bewal, and R. M. Gupta, "Probable vertical transmission of severe acute respiratory syndrome coronavirus 2 infection from mother to neonate," *Med J Armed Forces India*, vol. 77, no. Suppl 2, pp. S490–S493, Jul. **2021**, doi: 10.1016/j.mjafi.2020.11.026.
27. Kirtsman, M.; Diambomba, Y.; Poutanen, S.M.; Malinowski, A.K.; Vlachodimitropoulou, E.; Parks, W.T.; Erdman, L.; Morris, S.K.; Shah, P.S., "Probable congenital SARS-CoV-2 infection in a neonate born to a woman with active SARS-CoV-2 infection," *CMAJ*, vol. 192, no. 24, pp. E647–E650, Jun. **2020**, doi: 10.1503/cmaj.200821.
28. Von Kohorn, I.; Stein, S.R.; Shikani, B.T.; Ramos-Benitez, M.J.; Vannella, K.M.; Hewitt, S.M.; E Kleiner, D.; Alejo, J.C.; Burbelo, P.; I Cohen, J. et al., "In Utero Severe Acute Respiratory Syndrome Coronavirus 2 Infection," *J Pediatric Infect Dis Soc*, vol. 9, no. 6, pp. 769–771, Dec. **2020**, doi: 10.1093/jpids/piaa127.
29. Lesieur, E.; Torrents, J.; Fina, F.; Zandotti, C.; Blanc, J.; Collardeau-Frachon, S.; Gazin, C.; Sirgant, D.; Mezouar, S.; Otmani Idrissi, M.; et al., "Congenital Infection of Severe Acute Respiratory Syndrome Coronavirus 2 With Intrauterine Fetal Death: A Clinicopathological Study With Molecular Analysis," *Clin Infect Dis*, vol. 75, no. 1, pp. e1092–e1100, Aug. **2022**, doi: 10.1093/cid/ciab840.
30. Lorenz, N.; Treptow, A.; Schmidt, S.; Hofmann, R.; Raumer-Engler, M.; Heubner, G.; Gröber, K., "Neonatal Early-Onset Infection With SARS-CoV-2 in a Newborn Presenting With Encephalitic Symptoms," *Pediatr Infect Dis J*, vol. 39, no. 8, p. e212, Aug. **2020**, doi: 10.1097/INF.0000000000002735.
31. Marinho, P.S.; da Cunha, A.J.L.A.; Chimelli, L.; Avvad-Portari, E.; Andreiuolo, F.D.M.; de Oliveira-Szejnfeld, P.S.; Mendes, M.A.; Gomes, I.C.; Souza, L.R.Q.; Guimarães, M.Z.; et al., "Case Report: SARS-CoV-2 Mother-to-Child Transmission and Fetal Death Associated With Severe Placental Thromboembolism," *Front Med (Lausanne)*, vol. 8, p. 677001, **2021**, doi: 10.3389/fmed.2021.677001.
32. T. Marton, B. Hargitai, K. Hunter, M. Pugh, and P. Murray, "Massive Perivillous Fibrin Deposition and Chronic Histiocytic Intervillositis a Complication of SARS-CoV-2 Infection," *Pediatr Dev Pathol*, vol. 24, no. 5, pp. 450–454, Sep. **2021**, doi: 10.1177/10935266211020723.
33. Marzollo, R.; Aversa, S.; Prefumo, F.; Saccani, B.; Perez, C.R.; Sartori, E.; Motta, M., "Possible Coronavirus Disease 2019 Pandemic and Pregnancy: Vertical Transmission Is Not Excluded," *Pediatr Infect Dis J*, vol. 39, no. 9, pp. e261–e262, Sep. **2020**, doi: 10.1097/INF.0000000000002816.
34. Morales, H. S. G.; Cortés, D. V.; Hernández, H. S.; Guiot, M. L.; Torres, G. C. R.; Camacho, F. M. R.; Montoya, G. A.; Maldonado, B. F.; Bárcenas, J. G.; López, G. G. P., "Vertical transmission: evidence of COVID-19 in a twin pregnancy," *JBRA Assist Reprod*, vol. 26, no. 1, pp. 153–157, **2022**, doi: 10.5935/1518-0557.20210058.
35. O. M. Y. Leow, R. Aoyama, and S. M. Chan, "The evolution of severity of paediatric COVID-19 in Singapore: Vertical transmission and multisystem inflammatory syndrome in children," *Ann Acad Med Singap*, vol. 51, no. 2, pp. 115–118, Feb. **2022**, doi: 10.47102/annals-acadmedsg.2021426.

36. D. C. Ng, L. Chin, P. P. L. Choo, and U. Paramasivam, "COVID-19 in a premature infant," *BMJ Case Rep*, vol. 14, no. 5, p. e243783, May **2021**, doi: 10.1136/bcr-2021-243783.
37. Y. Parsa, N. Shokri, T. Jahedbozorgan, Z. Naeiji, S. Zadehmodares, and A. Moridi, "Possible Vertical Transmission of COVID-19 to the Newborn; a Case Report," *Arch Acad Emerg Med*, vol. 9, no. 1, p. e5, **2021**.
38. Popescu, D.E.; Cioca, A.; Muresan, C.; Navolan, D.; Gui, A.; Pop, O.; Marcovici, T.; Ilie, C.; Craina, M.; Boia, M.A., "A Case of COVID-19 Pregnancy Complicated with Hydrops Fetalis and Intrauterine Death," *Medicina (Kaunas)*, vol. 57, no. 7, p. 667, Jun. **2021**, doi: 10.3390/medicina57070667.
39. Pulinx, B.; Kieffer, D.; Michiels, I.; Petermans, S.; Strybol, D.; Delvaux, S.; Baldewijns, M.; Raymaekers, M.; Cartuyvels, R.; Maurissen, W., "Vertical transmission of SARS-CoV-2 infection and preterm birth," *Eur J Clin Microbiol Infect Dis*, vol. 39, no. 12, pp. 2441–2445, **2020**, doi: 10.1007/s10096-020-03964-y.
40. Reagan-Steiner, S.; Bhatnagar, J.; Martines, R.B.; Milligan, N.S.; Gisondo, C.; Williams, F.B.; Lee, E.; Estetter, L.; Bullock, H.; Goldsmith, C.S.; et al., "Detection of SARS-CoV-2 in Neonatal Autopsy Tissues and Placenta," *Emerg Infect Dis*, vol. 28, no. 3, pp. 510–517, Mar. **2022**, doi: 10.3201/eid2803.211735.
41. C. M. Rebello, L. P. Fascina, G. Annicchino, J. R. R. Pinho, R. de A. M. Yoshida, and R. S. B. Zacharias, "Vertical transmission of SARS-CoV-2 from infected pregnant mother to the neonate detected by cord blood real-time polymerase chain reaction (RT-PCR)," *Pediatr Res*, vol. 89, no. 7, pp. 1592–1593, May **2021**, doi: 10.1038/s41390-020-01193-9.
42. Richtmann, R.; Torloni, M.R.; Oyamada Otani, A.R.; Levi, J.E.; Crema Tobara, M.; de Almeida Silva, C.; Dias, L.; Miglioli-Galvão, L.; Martins Silva, P.; Macoto Kondo, M. et al., "Fetal deaths in pregnancies with SARS-CoV-2 infection in Brazil: A case series," *Case Rep Womens Health*, vol. 27, p. e00243, Jul. **2020**, doi: 10.1016/j.crwh.2020.e00243.
43. M. L. Rodrigues, G. Gasparinho, F. Sepúlveda, and T. Matos, "Signs suggestive of congenital SARS-CoV-2 infection with intrauterine fetal death: A case report," *Eur J Obstet Gynecol Reprod Biol*, vol. 256, pp. 508–509, Jan. 2021, doi: 10.1016/j.ejogrb.**2020**.11.042.
44. A. Sagara, M. Yamaguchi, Y. Mikami, T. Motohara, T. Ohba, and E. Kondoh, "Maternal thrombocytopenia precedes fetal death associated with COVID-19," *J Obstet Gynaecol Res*, vol. 48, no. 6, pp. 1475–1479, Jun. **2022**, doi: 10.1111/jog.15223.
45. Schwartz, D. A.; Baldewijns, M.; Benachi, A.; Bugatti, M.; Collins, R. R. J.; De Luca, D.; Facchetti, F.; Linn, R. L.; Marcelis, L.; Morotti, D.; et al., "Chronic Histiocytic Intervillositis With Trophoblast Necrosis Is a Risk Factor Associated With Placental Infection From Coronavirus Disease 2019 (COVID-19) and Intrauterine Maternal-Fetal Severe Acute Respiratory Syndrome Coronavirus 2 (SARS-CoV-2) Transmission in Live-Born and Stillborn Infants," *Arch Pathol Lab Med*, vol. 145, no. 5, pp. 517–528, May **2021**, doi: 10.5858/arpa.**2020**-0771-SA.
46. Sessa, R.; Masciullo, L.; Filardo, S.; Di Pietro, M.; Brandolino, G.; Brunelli, R.; Galoppi, P.; Terrin, G.; Viscardi, M.F.; Anastasi, E.; et al., "SARS-CoV-2 vertical transmission in a twin-pregnant woman: a case report," *Int J Infect Dis*, pp. S1201-9712(22)00558–6, Oct. **2022**, doi: 10.1016/j.ijid.2022.10.019.
47. Shaiba, L. A.; Hadid, A.; Altirkawi, K. A.; Bakheet, H. M.; Alherz, A. M.; Hussain, S. A.; Sobaih, B. H.; Alnemri, A. M.; Almaghrabi, R.; et al., "Case Report: Neonatal Multi-System Inflammatory Syndrome Associated With SARS-CoV-2 Exposure in Two Cases From Saudi Arabia," *Front Pediatr*, vol. 9, p. 652857, **2021**, doi: 10.3389/fped.2021.652857.
48. Shen,W.-B.; Turan, S.;Wang, B.; Cojocaru, L.; Harman, C.; Logue, J.; Reece, E.A.; Frieman, M.B.; Yang, P., "A SARS-CoV-2 Delta Variant Case Manifesting as Extensive Placental Infection and Fetal Transmission," *Gynecol Obstet Invest*, vol. 87, no. 2, pp. 165–172, **2022**, doi: 10.1159/000524905.
49. Shook, L. L.; Brigida, S.; Regan, J.; Flynn, J. P.; Mohammadi, A.; Etemad, B.; Siegel, M. R.; Clapp, M. A.; Li, J. Z.; Roberts, D. J.; Edlow, A. G., "SARS-CoV-2 Placentitis Associated With B.1.617.2 (Delta) Variant and Fetal Distress or Demise," *J Infect Dis*, vol. 225, no. 5, pp. 754–758, Jan. **2022**, doi: 10.1093/infdis/jiac008.
50. Sukhikh, G.; Petrova, U.; Prikhodko, A.; Starodubtseva, N.; Chingin, K.; Chen, H.; Bugrova, A.; Kononikhin, A.; Bourmenskaya, O.; Brzhozovskiy, A.; et al., "Vertical Transmission of SARS-CoV-2 in Second Trimester Associated with Severe Neonatal Pathology," *Viruses*, vol. 13, no. 3, p. 447, Mar. **2021**, doi: 10.3390/v13030447.
51. Vivanti, A.J.; Vauloup-Fellous, C.; Escourrou, G.; Rosenblatt, J.; Jouannic, J.M.; Laurent-Bellue, A.; De Luca, D "Factors associated with SARS-CoV-2 transplacental transmission," *Am J Obstet Gynecol*, vol. 227, no. 3, pp. 541-543.e11, Sep. **2022**, doi: 10.1016/j.ajog.2022.05.015.

52. Vivanti, A.J.; Vauloup-Fellous, C.; Prevot, S.; Zupan, V.; Suffee, C.; Do Cao, J.; Benachi, A.; De Luca, D "Transplacental transmission of SARS-CoV-2 infection," *Nat Commun*, vol. 11, no. 1, p. 3572, Jul. **2020**, doi: 10.1038/s41467-020-17436-6.
53. Zaigham, M.; Gisselsson, D.; Sand, A.; Wikström, A.-K.; von Wowern, E.; Schwartz, D. A.; Iorizzo, L.; Nelander, M.; Blomberg, M.; Papadogiannakis, N.; et al., "Clinical-pathological features in placentas of pregnancies with SARS-CoV-2 infection and adverse outcome: case series with and without congenital transmission," *BJOG*, vol. 129, no. 8, pp. 1361–1374, Jul. **2022**, doi: 10.1111/1471-0528.17132.
54. M. Zamaniyan, A. Ebadi, S. Aghajanpoor, Z. Rahmani, M. Haghshenas, and S. Azizi, "Preterm delivery, maternal death, and vertical transmission in a pregnant woman with COVID-19 infection," *Prenat Diagn*, vol. 40, no. 13, pp. 1759–1761, Dec. **2020**, doi: 10.1002/pd.5713.
55. P. Zhang, C. Salafia, T. Heyman, C. Salafia, S. Lederman, and B. Dygulska, "Detection of severe acute respiratory syndrome coronavirus 2 in placentas with pathology and vertical transmission," *Am J Obstet Gynecol MFM*, vol. 2, no. 4, p. 100197, Nov. **2020**, doi: 10.1016/j.ajogmf.2020.100197.
56. Zinserling, V. A.; Bornstein, S. R.; Narkevich, T. A.; Sukhanova, Y. V.; Semenova, N. Yu.; Vashukova, M. A.; Steenblock, C., "Stillborn child with diffuse SARS-CoV-2 viral infection of multiple organs," *IDCases*, vol. 26, p. e01328, Nov. **2021**, doi: 10.1016/j.idcr.2021.e01328.
57. C. K.-L. Lo, D. Mertz, and M. Loeb, "Newcastle-Ottawa Scale: comparing reviewers' to authors' assessments," *BMC Med Res Methodol*, vol. 14, p. 45, Apr. **2014**, doi: 10.1186/1471-2288-14-45.
58. Argueta, L. B.; Lacko, L. A.; Bram, Y.; Tada, T.; Carrau, L.; Rendeiro, A. F.; Zhang, T.; Uhl, S.; Lubor, B. C.; Chandar, V.; Gil, C.; Zhang, W., "Inflammatory responses in the placenta upon SARS-CoV-2 infection late in pregnancy," *iScience*, vol. 25, no. 5, p. 104223, May **2022**, doi: 10.1016/j.isci.2022.104223.
59. Fenizia, C.; Biasin, M.; Cetin, I.; Vergani, P.; Mileto, D.; Spinillo, A.; Gismondo, M.R.; Perotti, F.; Callegari, C.; Mancon, A.; et al., "Analysis of SARS-CoV-2 vertical transmission during pregnancy," *Nat Commun*, vol. 11, no. 1, p. 5128, Oct. **2020**, doi: 10.1038/s41467-020-18933-4.
60. Garrido-Pontnou, M.; Navarro, A.; Camacho, J.; Crispi, F.; Alguacil-Guillén, M.; Moreno-Baró, A.; Hernandez-Losa, J.; Sesé, M.; Ramón y Cajal, S.; et al., "Diffuse trophoblast damage is the hallmark of SARS-CoV-2-associated fetal demise," *Mod Pathol*, vol. 34, no. 9, Art. no. 9, Sep. **2021**, doi: 10.1038/s41379-021-00827-5.
